# Alcohol and Sedative Use Disorders in the Lebanese Population: Role of Sleep and Psychiatric Factors

**DOI:** 10.1192/j.eurpsy.2024.305

**Published:** 2024-08-27

**Authors:** K. Chamoun, N. Wehbe, P. Salameh, H. Sacre, M. Atallah, J. Mouawad, L. Rabbaa, B. Megarbane, A. Hajj

**Affiliations:** ^1^Laboratoire de Pharmacologie, Pharmacie Clinique et Contrôle de Qualité des Médicaments; ^2^Faculty of Pharmacy, Saint Joseph University, Beirut, Lebanon; ^3^Inserm, UMR-S1144, Université Paris Cité, Paris, France; ^4^INSPECT-LB (Institut National de Santé Publique, d’Épidémiologie Clinique et de Toxicologie-Liban), Beirut; ^5^School of Medicine, Lebanese American University, Byblos, Lebanon; ^6^Department of Primary Care and Population Health, University of Nicosia Medical School, Nicosia, Cyprus; ^7^Faculty of Pharmacy, Lebanese University, Hadat, Lebanon; ^8^Faculty of Pharmacy, Université Laval; ^9^Oncology Division, CHU de Québec- Université Laval Research Center, Quebec, Canada

## Abstract

**Introduction:**

Alcohol and sedative substance use disorders are escalating global public health challenges. Lebanon has grappled with multiple crises, including economic, healthcare, and social issues.

**Objectives:**

This study aimed to assess the correlates of the alcohol and sedative substance use risk scores with sociodemographic and clinical factors, including sleep disorders, chronotype, anxiety, and depression.

**Methods:**

A cross-sectional study was conducted among the Lebanese population using several validated scales to assess the risk of alcohol and sedative substance use, including the Alcohol, Smoking, and Substance Involvement Screening Test (ASSIST). Other tools evaluated chronotype, sleep, and mood disturbances. Bivariate and multivariable analyses were then performed, taking the alcohol and sedative scores as dependent variables.

**Results:**

A total of 646 participants were included. Multivariate analysis revealed positive and significant correlations between higher ASSIST-alcohol scores and personal history of alcohol abuse (B=4.61), family history of prescription substance abuse (B=1.763), psychiatric disorders (B=2.898), and worse Insomnia Severity Index scores (Beta=0.14). Conversely, ASSIST-alcohol scores negatively correlated with weight (B= -0.39) and morning chronotype (B=-0.084). Positive correlations were identified between higher ASSIST-alcohol scores and personal history of illicit substance abuse (B=2.834), prescription substance abuse (B=2.252), sleep quality (B=0.130), and sleep severity (B=0.082), while negatively correlating with cigarette smoking (B=-0.038).

**Conclusions:**

This study elucidates the role of several predisposing factors to alcohol and sedative use disorders in Lebanon, including history of substance abuse, psychiatric disorders, sleep disorders, and chronotype. These findings advocate, in particular, for the integration of sleep disorder assessment and management into addiction rehabilitation programs.

**Disclosure of Interest:**

None Declared

